# Analysis and Molecular Determinants of HIV RNase H Cleavage Specificity at the PPT/U3 Junction

**DOI:** 10.3390/v13010131

**Published:** 2021-01-18

**Authors:** Mar Álvarez, Enrique Sapena-Ventura, Joanna Luczkowiak, Samara Martín-Alonso, Luis Menéndez-Arias

**Affiliations:** Centro de Biología Molecular Severo Ochoa (Consejo Superior de Investigaciones Científicas & Universidad Autónoma de Madrid), Campus de Cantoblanco-UAM, 28049 Madrid, Spain; malvarez@cbm.csic.es (M.Á.); sapenaventura@gmail.com (E.S.-V.); asialuczkowiak@interia.pl (J.L.); samara.martin@cbm.csic.es (S.M.-A.)

**Keywords:** HIV, reverse transcriptase, antiretroviral drug resistance, DNA synthesis, doravirine, RNase H

## Abstract

HIV reverse transcriptases (RTs) convert viral genomic RNA into double-stranded DNA. During reverse transcription, polypurine tracts (PPTs) resilient to RNase H cleavage are used as primers for plus-strand DNA synthesis. Nonnucleoside RT inhibitors (NNRTIs) can interfere with the initiation of plus-strand DNA synthesis by enhancing PPT removal, while HIV RT connection subdomain mutations N348I and N348I/T369I mitigate this effect by altering RNase H cleavage specificity. Now, we demonstrate that among approved nonnucleoside RT inhibitors (NNRTIs), nevirapine and doravirine show the largest effects. The combination N348I/T369I in HIV-1_BH10_ RT has a dominant effect on the RNase H cleavage specificity at the PPT/U3 site. Biochemical studies showed that wild-type HIV-1 and HIV-2 RTs were able to process efficiently and accurately all tested HIV PPT sequences. However, the cleavage accuracy at the PPT/U3 junction shown by the HIV-2_EHO_ RT was further improved after substituting the sequence YQEPFKNLKT of HIV-1_BH10_ RT (positions 342–351) for the equivalent residues of the HIV-2 enzyme (HQGDKILKV). Our results highlight the role of β-sheets 17 and 18 and their connecting loop (residues 342–350) in the connection subdomain of the large subunit, in determining the RNase H cleavage window of HIV RTs.

## 1. Introduction

In retroviruses, reverse transcription is a relatively complex process involving the conversion of single-stranded viral genomic RNA into double-stranded DNA that integrates into the host cell chromosome. Reverse transcriptases (RTs) synthesize minus-strand DNA using viral genomic RNA as a template (RNA-dependent DNA polymerase activity) while the RT’s ribonuclease H (RNase H) nucleolytic activity degrades the RNA strand in RNA/DNA hybrids. For the synthesis of plus-strand DNA, retroviral RTs use minus-strand DNA as a template (DNA-dependent DNA polymerase activity). A cellular tRNA primer that binds near the 5′ end of the viral genome acts as a primer in minus-strand DNA synthesis, while RNA polypurine tracts (PPTs) resilient to cleavage by the RT’s RNase H activity serve as primers for plus-strand DNA synthesis [1,2].

**Figure 1:**
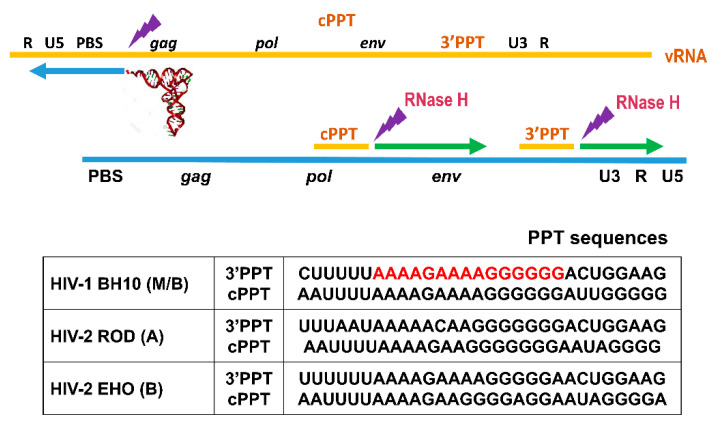
HIV reverse transcription and PPT sequences. The upper panel shows the initiation of reverse transcription with the viral RNA (vRNA) in orange, and the minus-strand DNA in blue. The relative location of viral genes (*gag*, *pol* and *env*), and relevant sequences (R, U5, PBS, cPPT, 3′PPT and U3) are shown above the viral RNA. The plus-strand DNA strand is shown in green. Polypurine tracts (cPPT and 3′PPT) are used as primers and lightning symbols indicate RNase H cleavage sites. The lower panel shows PPT sequences of HIV-1_BH10_, HIV-2_ROD_ and HIV-2_EHO_, taken from GenBank accession numbers AH002345.2, X05291 and U27200, respectively. The 3′PPT of HIV-1_BH10_ is highlighted in red.

All retroviruses have PPT sequences at the 3′ end of the viral genome, located at the PPT/U3 junction (3′PPT). In human immunodeficiency viruses (HIV) and other lentiviruses, there is a second PPT near the center of the viral genome, within the integrase-coding sequence of the *pol* gene, designated as cPPT (Figure 1). During reverse transcription, this cPPT facilitates the formation of a triple DNA structure that appears to be essential for importing the pre-integration complex into the nucleus of non-dividing cells [3,4]. HIV-1 PPTs contain 15 ribonucleotides, comprising a stretch of eight adenines with a single intervening guanine and a final homopolymeric sequence of six guanines. The 5′ end of the PPTs is flanked by uridine-rich regions that, together with the intervening guanine, evolved to protect the integrity of the downstream PPT sequence [5].

The tRNA and PPT primers are removed by the RNase H activity of the RT after plus-strand strong-stop DNA synthesis. Specific and accurate RNase H cleavage is important for viral replication since the ends of the linear viral DNA must be suitable for integration into the host cell genome [6]. Structural and biochemical studies of HIV-1 RT have shown that the PPT sequence as well as amino acid residues in the RNase H domain of the enzyme (e.g., Gln475 and Tyr501) are important for defining RNase H cleavage specificity [7,8,9].

HIV RTs are heterodimeric enzymes composed of subunits of 66 and 51 kDa in the case of HIV-1, and 68 and 54 kDa in the case of HIV-2 [2,10]. Both enzymatic activities (DNA polymerase and RNase H) reside on the large subunit of the RT. Asp110, Asp185 and Asp186 form the catalytic triad in the polymerase active site, whereas Asp443, Glu478, Asp498 and Asp549 are the RNase H catalytic residues (Asp442, Glu477, Asp497 and Asp548 in HIV-2 RT, due to the deletion of one amino acid in the connection subdomain of the enzyme). Both active sites are separated by a distance equivalent to a double helix of 18–20 base pairs ([11]; for a recent review, see ref. [12]), and coordination between both activities is essential for reverse transcription. Pre-steady-state kinetics [13] combined with the analysis of RNase H cleavages obtained with cross-linked complexes of HIV-1 RT and RNA/DNA hybrids [14] showed that RNase H hydrolysis occurs at a six-fold reduced rate compared to nucleotide incorporation, implying a processing periodicity of about 6–7 nucleotides. However, the sequence context influences both DNA polymerization and RNase H hydrolysis rates, resulting in an uneven distribution of RNase H cleavage sites along the viral RNA sequence. RNase H cleavage efficiency can be of utmost importance for PPT removal due to the reduced rate of nucleotide incorporation observed at the initiation of plus-strand DNA synthesis [15,16]. Thus, slow DNA polymerization favors a longer exposure of the PPT sequence to the RNase H catalytic site.

In a previous study, we showed that amino acid substitutions in the connection subdomain of HIV-1 RT such as N348I and T369I affect the rigidity of a hypothetical hinge connecting the DNA polymerase (residues 1–330) and the RNase H (residues 440–560) domains in the 66-kDa subunit of HIV-1 RT [17]. PPT removal by the double-mutant N348I/T369I RT was rather inefficient since this enzyme had an impaired ability to produce short RNA products. In addition, N348I, T369I and A376S are amino acid changes that confer resistance to nonnucleoside RT inhibitors (NNRTIs) such as nevirapine, delavirdine and efavirenz [18,19], despite being located away from the NNRTI binding site (reviewed in refs. [20,21]). N348I and A376S were found with increased prevalence in nevirapine-treated patients with a higher risk of virological failure [22,23]. Several studies have shown that NNRTIs such as nevirapine or efavirenz can modulate RNase H activity through long-range interactions that depend on the structure of the RNA/DNA hybrid [24,25,26,27,28,29]. Furthermore, Biondi et al. showed that the wild-type (WT) HIV-1 RT’s RNase H had increased PPT cleavage efficiency in reactions carried out in the presence of nevirapine and efavirenz [30]. Similar effects were also reported for mutant RTs bearing single amino-acid substitutions in the connection subdomain such as T369I, T369V and T376S, and selected in patients treated with NNRTIs. N348I/T369I and to a lesser extent N348I interfere with plus-strand DNA synthesis by reducing PPT removal efficiency in the presence of nevirapine and efavirenz [17]. However, nevirapine had no effect on PPT removal in reactions carried out with HIV-1 RT containing the drug resistance-associated substitution K103N [17].

We now extended the analysis of PPT cleavage efficiency and specificity to additional mutations found with increased prevalence in patients treated with NNRTIs, including connection subdomain mutations at positions 399 and 400 [19,31,32,33,34]. In addition, we compared cleavage patterns obtained with template-primer complexes containing representative PPT sequences and prototypic WT HIV RTs of four major clades: (i) HIV-1_BH10_ (group M-subtype B); (ii) HIV-1_ESP49_ (group O); (iii) HIV-2_ROD_ (group A); and (iv) HIV-2_EHO_ (group B). These enzymes show a relatively high number of amino acid sequence differences in their connection subdomain around residue 348, including deletion of one amino acid residue found in HIV-2 RT, as compared with HIV-1 RTs. PPT cleavage patterns obtained with connection subdomain mutants of HIV-2_EHO_ RT underscore the importance of the amino acid sequence around position 348 in controlling the RNase H cleavage window and support its functional role in the initiation of plus-strand DNA synthesis.

## 2. Materials and Methods

### 2.1. Expression and Purification of Recombinant RTs

WT HIV-1_BH10_ RT and mutants N348I and N348I/T369I [17] and HIV-1 group O RTs (WT HIV-1_ESP49_ RT and mutant T355A/Q357M/K358R/A359G/S360A (5M)) [35,36] were obtained as heterodimers composed of subunits of 66 and 51 kDa, containing poly-histidine tags at the C-terminus of p66 [37,38]. WT HIV-2_ROD_ RT was also obtained as a heterodimeric enzyme (p68/p54) after co-expression of the p68-coding region of HIV-2_ROD_ together with the HIV-2_D194_ protease, using constructs derived from plasmid pT5m [39]. WT HIV-2_EHO_ RT was expressed in *E. coli* XL1 Blue, using plasmids p66RTB [35] and pATPR [37] encoding the large HIV-2_EHO_ RT subunit and the viral protease, respectively. The codon-optimized HIV-2_EHO_ p68-coding sequence (GenBank accession number U27200) with flanking EcoRI and XhoI sites was obtained from GenScript (Piscataway, NJ, USA), and cloned into the p66RTB plasmid. Heterodimeric p68/p54 HIV-2_EHO_ RT with a poly-histidine tag at the C-terminus of p68 was purified as described for HIV-2_ROD_ RT by immobilized metal affinity chromatography on Ni^2+^-nitriloacetic acid agarose, followed by ion-exchange chromatography [39]. Some enzymes were further purified by heparin-Sepharose (GE Healthcare) affinity chromatography, as previously described [40]. RTs were quantified by active site titration before biochemical studies, using template-primer D38/25PGA [38,41].

### 2.2. Mutagenesis

Site-directed mutagenesis was carried out using the standard QuikChange^TM^ protocol (Stratagene). Plasmids p66RTB containing the p66-coding sequence of HIV-1_BH10_ and the codon-optimized p68-coding sequence of HIV-2_EHO_ were used as templates. Complementary mutagenic primers (Supplementary Table S1) were used to amplify entire plasmids in a thermocycling reaction carried out with high-fidelity Pfu DNA polymerase. Double mutants E138K/M184I and E138K/M184V were prepared from the single-mutant E138K HIV-1 RT, previously described [17]. Quadruple mutants E138K/M184I/N348I/T369I and E138K/M184V/N348I/T369I were obtained after introducing mutations E138K and M184I/V in sequential order, using as template the plasmid p66RTB containing the p66-coding sequence of mutant N348I/T369I HIV-1_BH10_ RT. Mutant G344E/D345P/ins346F of HIV-2_EHO_ RT was prepared with mutagenic oligonucleotides containing the nucleotide changes required to introduce G344E and D345P substitutions in the presence of the insertion of Phe346, and the template RT-coding region encoding the HIV-2_EHO_ RT with the 346F insertion alone. The mutant H342Y/G344E/D345P/ins346F/V351T was derived from mutant G344E/D345P/ins346F, after introducing mutations H342Y and V351T in sequential order with the mutagenic primers indicated in the Supplementary Table S1. After mutagenesis, RT-coding regions were entirely sequenced and, if correct, used for RT expression and purification.

### 2.3. Nucleotides, Template-Primers and Antiretroviral Drugs

Stock solutions (100 mM) of dNTPs were obtained from GE Healthcare. [γ−^32^P] ATP (10 mCi/mL; 3000 Ci/mmol) was provided by Perkin Elmer. Synthetic oligonucleotides were obtained from Sigma and Integrated DNA Technologies. Nevirapine, efavirenz, etravirine and rilpivirine were obtained from the AIDS Research and Reference Reagent Program, Division of AIDS, NIAID, NIH. Rilpivirine and etravirine were supplied to the Program by Tibotec Pharmaceuticals, Inc. Doravirine, raltegravir (potassium salt) and dolutegravir were purchased from MedChem Express LLC (Monmouth Junction, NJ, USA).

### 2.4. RNase H Activity Assays

Assays were carried out in the presence or absence of antiretroviral drugs in 50 mM Tris-HCl pH 8.0 buffer, containing 50 mM NaCl, 5 mM MgCl_2_ and 1% DMSO [17,42]. Template-primer concentrations were usually in the range 25–50 nM, and RT concentrations varied between 25 and 125 nM, depending on the assays. The effects of antiretroviral drugs on PPT cleavage were determined after incubating the RT and varying concentrations of the inhibitors for 5 min at room temperature, and then adding the template-primer. After a 10-min incubation at 37 °C, reactions were initiated by adding MgCl_2_. Aliquots were removed at appropriate times (usually between 20 s and 3 min) and quenched with an equal volume of stop solution (10 mM EDTA in 90% formamide containing 3 mg/mL xylene cyanol FF and 3 mg/mL bromophenol blue). In assays carried out under single-turnover conditions, RTs and template-primers were pre-incubated in 50 mM Tris-HCl pH 8.0 containing 50 mM NaCl for 5 min at 37 °C, and reactions were then initiated by adding MgCl_2_ and sodium heparin to final concentrations of 5 mM and 3 mg/mL, respectively. A two- to five-fold excess of enzyme over template-primer was used in all experiments. Products were analyzed after heating at 90 °C for 10 min by denaturing polyacrylamide gel electrophoresis and quantified with a BAS 1500 scanner (Fuji) using the program TINA version 2.09 (Raytest Isotopenmessgerate Gmbh, Staubenhardt, Germany).

## 3. Results

### 3.1. Effects of NNRTIs and Integrase Inhibitors on PPT Removal by HIV-1_BH10_ RT

**Figure 2:**
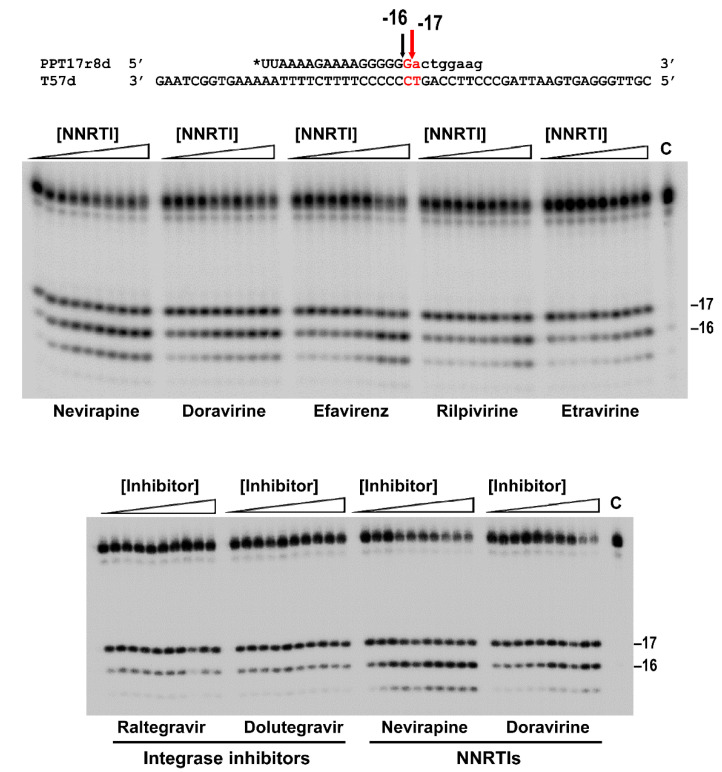
Cleavage of the chimeric PPT(RNA)-DNA oligonucleotide in the T57d/PPT17r8d complex in the presence of increasing concentrations of approved antiretroviral drugs. Assays were carried out with increasing concentrations of NNRTIs or integrase inhibitors: 0, 0.08, 0.16, 0.3125, 0.625, 1.25, 2.5, 5, 10 and 20 μM. Template-primer and WT HIV-1_BH10_ RT concentrations in these assays were 25 and 125 nM, respectively. Aliquots were withdrawn after a 20 s-incubation. C stands for control (uncleaved PPT17r8d primer). Images show representative gels obtained from at least three independent experiments. Arrow and text in red indicate the location of the PPT/U3 cleavage site.

During reverse transcription, initiation of plus-strand DNA synthesis is particularly sensitive to NNRTI inhibition [16]. Nevirapine and other NNRTIs enhance RNase H-mediated PPT removal [17,30]. Thus, in the presence of nevirapine, initiation of plus-strand DNA synthesis could be impaired due to the reduced amounts of PPT primer available. Previously, we showed that PPT removal was enhanced by nevirapine and efavirenz, while rilpivirine had a relatively small effect on RNase H-mediated PPT cleavage [17]. In those experiments, we used a template-primer mimicking the initial steps of plus-strand DNA synthesis. This substrate was a chimeric RNA-DNA primer (PPT17r8d) that contained the 3′PPT RNA oligonucleotide sequence of HIV-1_BH10_ (17 nucleotides) with an 8-nucleotide extension of DNA, annealed to a complementary DNA template of 57 nucleotides (T57d). Using this T57d/PPT17r8d duplex (Figure 2), we extended the analysis to etravirine and the recently approved NNRTI doravirine. As shown in Figure 2, the cleavage patterns observed with etravirine were very similar to those obtained with rilpivirine. Significant trimming of the PPT (i.e., cleavage at position −16) was observed only at the highest tested concentrations (10 and 20 µM), while in the case of nevirapine, similar effects were observed at much lower concentrations (i.e., around 160 nM). Interestingly, doravirine was also a good enhancer of RNase H-mediated cleavage of the PPT (Figure 2). To facilitate the comparison, assay conditions were chosen to ensure that the reaction products shown on the gels were obtained in the linear phase for most of the drug concentrations analyzed.

**Figure 3:**
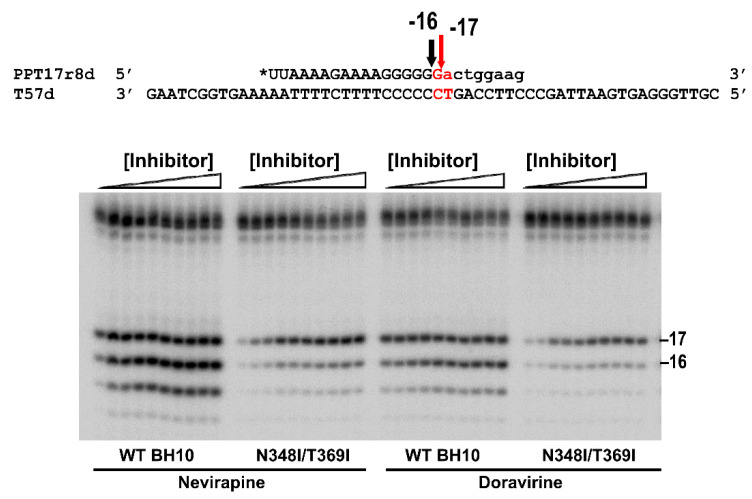
Cleavage of the chimeric PPT(RNA)-DNA oligonucleotide in the T57d/PPT17r8d complex in the presence of increasing concentrations of nevirapine and doravirine by WT HIV-1_BH10_ RT and mutant N348I/T369I. In each experiment the NNRTI concentrations used were 0, 0.08, 0.16, 0.3125, 0.625, 1.25, 2.5, 5, 10 and 20 μM. Template-primer and RT concentrations in these assays were 25 and 125 nM, respectively. Aliquots were withdrawn after a 20 s-incubation. Experiments were carried out twice and a representative gel is shown. Arrow and text in red indicate the location of the PPT/U3 cleavage site.

Cleavage patterns obtained with doravirine were similar to those observed in reactions carried out with nevirapine, although amounts of shorter products were smaller in the case of doravirine. The stimulatory effect of efavirenz was higher at micromolar concentrations of the inhibitor, but less pronounced at lower concentrations tested. Unlike in the case of NNRTIs, raltegravir and dolutegravir (integrase inhibitors used as controls in these experiments) had no effect on the PPT removal efficiency of the WT HIV-1_BH10_ RT (Figure 2). As previously shown for nevirapine [17], amino acid substitutions N348I/T369I reduced the efficiency of the WT enzyme in PPT cleavage assays carried out in the presence of doravirine (Figure 3), suggesting that those mutations could also contribute to doravirine resistance, despite being located away from the NNRTI binding site. These results are consistent with the reduced RNase H activity shown by N348I and N348I/T369I HIV-1 RTs in assays carried out with different RNA/DNA hybrids, with or without PPTs [17], and suggest that the observed NNRTI effects could be attributed to a different positioning of the template-primer within the nucleic acid binding cleft.

### 3.2. Dominant Effects of N348I/T369I on the RNase H Cleavage Window at the PPT/U3 Junction

**Figure 4:**
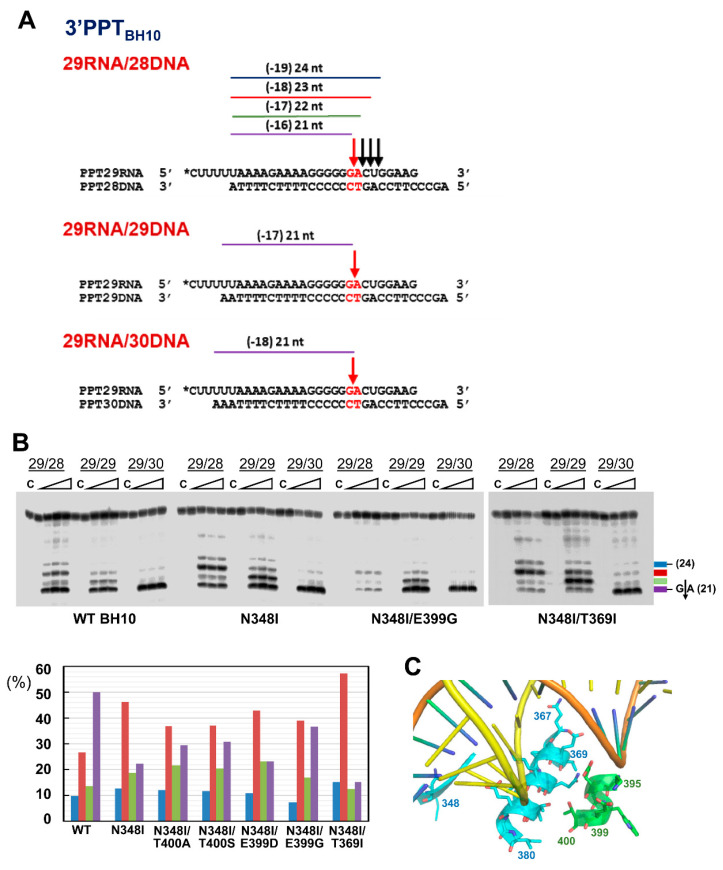
Effect of HIV-1_BH10_ RT connection subdomain mutations on the RNase H cleavage window. (**A**) Template-primers used in the assay and major cleavage sites observed. Arrows and texts in red indicate the location of the PPT/U3 cleavage site in the RNA/DNA complexes used. (**B**) Reactions were carried out in the presence of RT and template-primer at concentrations of 125 nM and 25 nM, respectively. RT and template-primer were pre-incubated for 5 min at 37 °C, and aliquots were withdrawn at 0, 20, 40 and 180 s after the addition of magnesium and heparin. Lane C shows control experiments including heparin in the pre-incubation buffer. Numbers above indicate the template-primer used in each experiment: 29/28, 29/29 and 29/30 for 29RNA/28DNA, 29RNA/29DNA and 29RNA/30DNA hybrids, respectively. Histograms below indicate the relative proportions of hydrolysis products obtained with mutant RTs and the 29RNA/28DNA complex and were obtained from at least three independent experiments. Less than 5% inter-assay variabilities were observed in these assays. Blue, red, green and purple bars represent the relative amounts of RNase H cleavage products of 24, 23, 22 and 21 nucleotides, respectively. (**C**) WT HIV-1 RT structure of the connection subdomain showing the location of relevant residues in p66 (Asn348, Thr369) and p51 (Glu399, Thr400). The template strand is shown in yellow, while the primer strand is shown in orange. The image was prepared using Pymol and the Protein Data Bank structure 6UIS.

Suppression of the NNRTI-mediated increase of PPT cleavage efficiency by N348I HIV-1_BH10_ RT and the double-mutant N348I/T369I has been associated with their reduced capacity to generate short RNA products [17]. These effects were demonstrated through the analysis of the RNase H cleavage window specificity, using RNA/DNA template-primers containing the HIV-1_BH10_ 3′PPT sequence and recessed 3′ ends (Figure 4A). As shown in control experiments carried out with 29RNA/28DNA, 29RNA/29DNA and 29RNA/30DNA hybrids and the N348I/T369I RT (Figure 4B, right panel), the shortest RNA products rendering the correct cleavage at the PPT/U3 junction were obtained with the 29RNA/30DNA template-primer and corresponded to an RNase H cleavage window of 18 nucleotides. However, the N348I/T369I enzyme was unable to cleave efficiently the substrate at the correct G*A site when the distance to the 3′ end of the DNA was reduced to 16 nucleotides, as in template-primer 29RNA/28RNA. This pattern was rather different from the one obtained with WT HIV-1_BH10_ RT, whose cleavage efficiency at the PPT/U3 junction was high for all three tested RNA/DNA complexes. Interestingly, N348I alone showed an intermediate phenotype with partial cleavage at the G*A when the 29RNA/28DNA hybrid was used (Figure 4B).

Crystal structures of HIV-1 RT showed that neither Asn348 nor Thr369 interacts with the RNA/DNA hybrid [29,43,44]. However, the conformation of the hybrid within the nucleic acid binding cleft could be affected by interactions between the side chain of Thr369 in α-helix L of p66 and residues of α-helix L in p51 (Figure 4C). This α-helix includes residues 394–404. Among them, E399D or G and T400A or S are known polymorphisms whose prevalence increases in patients treated with NNRTIs [19,31,32,33,34]. We tested whether those amino acid substitutions could affect the RNase H PPT cleavage window in the presence of N348I in a similar manner as T369I. Histograms in Figure 4B show that the amino acid substitution E399D had negligible effects on the cleavage patterns obtained with template-primer 29RNA/28DNA. However, the addition of E399G, T400A or T400S to N348I RT produced a slight increase in the amount of correctly processed PPT sequence, suggesting that those mutations could revert in part the RNase H cleavage window defect shown by N348I RT.

**Figure 5:**
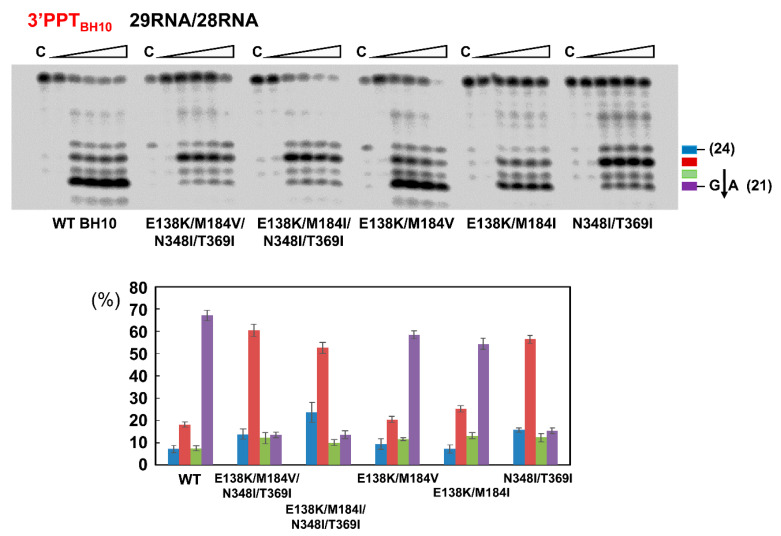
Effect of drug resistance mutations E138K and M184V or M184I on the RNase H cleavage window of WT and mutant N348I/T369I HIV-1_BH10_ RT. Reactions were carried out in the presence of RT and template-primer 29RNA/28DNA (see Figure 4A) at concentrations of 125 nM and 25 nM, respectively. RT and 29RNA/28DNA were pre-incubated for 5 min at 37 °C, and aliquots were withdrawn at 0, 20, 40, 60 and 180 s after the addition of magnesium and heparin. Lane C shows control experiments including heparin in the pre-incubation buffer. Histograms indicate the proportions of hydrolysis products obtained from at least three independent experiments. Blue, red, green and purple bars represent the relative amounts of RNase H cleavage products of 24, 23, 22 and 21 nucleotides, respectively. The 21-nucleotide product derives from cleavage at the PPT/U3 cleavage site (indicated with an arrow in the top panel).

Additional analyses carried out with rilpivirine resistance-associated mutations E138K/M184I or E138K/M184V showed that the combination of amino acid substitutions N348I and T369I had a dominant effect on the RNase H cleavage patterns observed with the PPT-containing 29RNA/28DNA template-primer complex (Figure 5). While preferential cleavage at the G*A site of the PPT/U3 junction was observed with mutants E138K/M184I and E138K/M184V and the WT HIV-1_BH10_ RT, the presence of N348I/T369I in any of those sequence contexts altered the cleavage preferences and impaired correct processing of the PPT.

### 3.3. RNase H Cleavage Patterns of Prototypic HIV RTs and Representative PPTs

**Figure 6:**
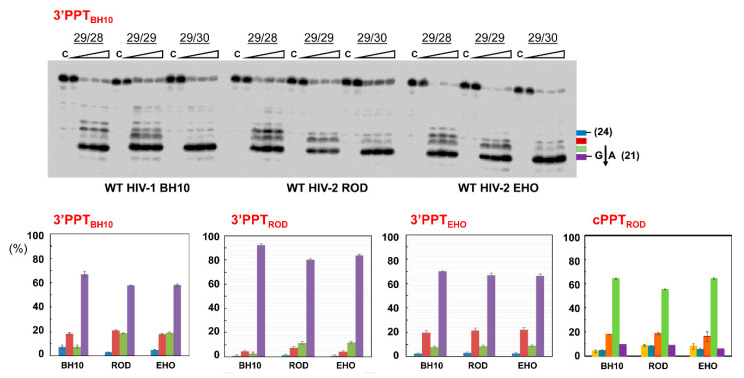
RNase H cleavage specificity of WT HIV-1_BH10_, HIV-2_ROD_ and HIV-2_EHO_ RTs on representative PPTs. Reactions were carried out in the presence of RT and template-primer at concentrations of 125 nM and 25 nM, respectively. RT and template-primer were pre-incubated for 5 min at 37 °C, and aliquots were withdrawn at 0, 20, 40 and 180 s after the addition of magnesium and heparin. Lane C shows control experiments including heparin in the pre-incubation buffer. Numbers above indicate the template-primer used in each experiment: 29/28, 29/29 and 29/30 for 29RNA/28DNA, 29RNA/29DNA and 29RNA/30DNA hybrids, respectively (see Figure 4A for sequence details). The 21-nucleotide products that derive from cleavage at the PPT/U3 cleavage site when using the 3’PPT_BH10_ complexes are indicated with an arrow in the right side of the gel. Histograms below indicate the proportions of hydrolysis products obtained with the three WT RTs and 29RNA/28DNA complexes representing the indicated PPTs (see Figure 4A and Supplementary Figure S1 for sequence details). Bar colors indicate the size of the oligonucleotide products: 21 (purple), 22 (green), 23 (red), 24 (blue) and 25 (yellow). Represented values were obtained from at least three independent determinations.

The studies described above were carried out with HIV-1_BH10_ RT variants and template-primers mimicking the 3′PPT sequence found in the BH10 strain of HIV-1. We extended this analysis to compare the specificity of WT HIV-1_BH10_ RT and WT RTs of phylogenetically distinct HIV-2 strains ROD and EHO, representing group A and B clades respectively, using template-primers containing PPTs of those viruses (sequences given in Supplementary Figure S1). The results shown in Figure 6 revealed that all 3′PPTs were efficiently processed by HIV-1 and HIV-2 RTs, even with a more restrictive 29RNA/28DNA template-primer. Accurate processing at the PPT/U3 junction was more efficient for HIV-2 PPTs than for the 3′PPT of HIV-1_BH10_. In all cases, more than 60% of the hydrolysis products corresponded to the oligonucleotide obtained after cleavage of the 29RNA at the G*A site of 3′PPT_BH10_ and 3′PPT_ROD_, or the A*A site in the PPT/U3 junction of HIV-2_EHO_ (sequences and cleavage sites are shown in Supplementary Materials (Figures S1 and S2). Interestingly, we also observed small differences in the cleavage patterns obtained with WT HIV-2_ROD_ and HIV-2_EHO_ RTs as compared with WT HIV-1_BH10_ RT.

Despite being relatively small, differences between HIV-1 and HIV-2 enzymes were detectable with substrates containing 3′PPTs of HIV-1_BH10_ and HIV-2_ROD_ strains (Figure 6, and Supplementary Figure S2). In contrast, RNase H cleavage patterns were almost identical for all three enzymes when using hybrids containing the 3′PPT_EHO_ and cPPT_ROD_ sequences. In the case of the cPPT_ROD_ hybrids, cleavage at the G*A site (corresponding to the 21-nt product, and represented in purple in the histogram) was rather inefficient, particularly when the 29RNA/28DNA was used (Supplementary Figure S2). Tested RTs showed a marked preference for cleavage at the adjacent A*A site, as expected if we assume a canonical length of 15 nucleotides for HIV PPTs.

It should be noted that the PPT tract of the cPPT of HIV-1_BH10_ is identical to that of the 3′PPT although some differences can be found at flanking sequences (Figure 1). In addition, the 3′PPT of HIV-2_ROD_ contains an intervening cytidine that breaks the integrity of the PPT, although both PPTs show limited variability among HIV-1 and HIV-2. In any case, our results underline the robust and consistent cleavage patterns obtained with the different PPTs analyzed.

**Figure 7:**
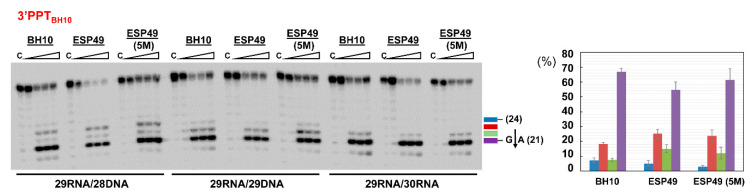
RNase H cleavage specificity of group M and group O HIV-1 RTs. Reactions were carried out in the presence of RT and template-primer at concentrations of 125 nM and 25 nM, respectively. RT and template-primer were pre-incubated for 5 min at 37 °C, and aliquots were withdrawn at 0, 20, 40 and 180 s after the addition of magnesium and heparin. Lane C shows control experiments including heparin in the pre-incubation buffer. Numbers above indicate the template-primer used in each experiment: 29/28, 29/29 and 29/30 for 29RNA/28DNA, 29RNA/29DNA and 29RNA/30DNA hybrids, respectively (see Figure 4A for sequence details). The histogram on the right panel shows the relative amounts of hydrolysis products obtained with template-primer 29RNA/28DNA, representing the 3′PPT_BH10_ sequence. Represented values were obtained from at least three independent determinations. Blue, red, green and purple bars represent the relative amounts of RNase H cleavage products of 24, 23, 22 and 21 nucleotides, respectively. The 21-nucleotide product derives from cleavage at the PPT/U3 cleavage site (indicated with an arrow in the left panel).

Cleavage patterns obtained with WT RT and the 3′PPT substrates of HIV-1_BH10_ were similar to those obtained with WT HIV-1_ESP49_, a prototypic group O RT, with 3′PPT_BH10_ duplexes (Figure 7). In both cases, the 23-nt product corresponding to the C*U cleavage outside the PPT was the most abundant secondary product in reactions carried out with the 29RNA/28DNA template-primer. Although our studies suggest that connection subdomain mutations could affect PPT cleavage efficiency, we found no differences with the WT enzyme in assays carried out with an HIV-1_ESP49_ RT containing five amino acid substitutions in the connection subdomain (T355A/Q357M/K358R/A359G/S360A (5M)). Our previously published studies had shown the higher template-primer affinity of this enzyme, in comparison with the WT HIV-1_ESP49_ RT [36].

### 3.4. Characterization of Connection Subdomain Mutants of HIV-2_EHO_ RT and Their Effect on PPT Processing

**Figure 8:**
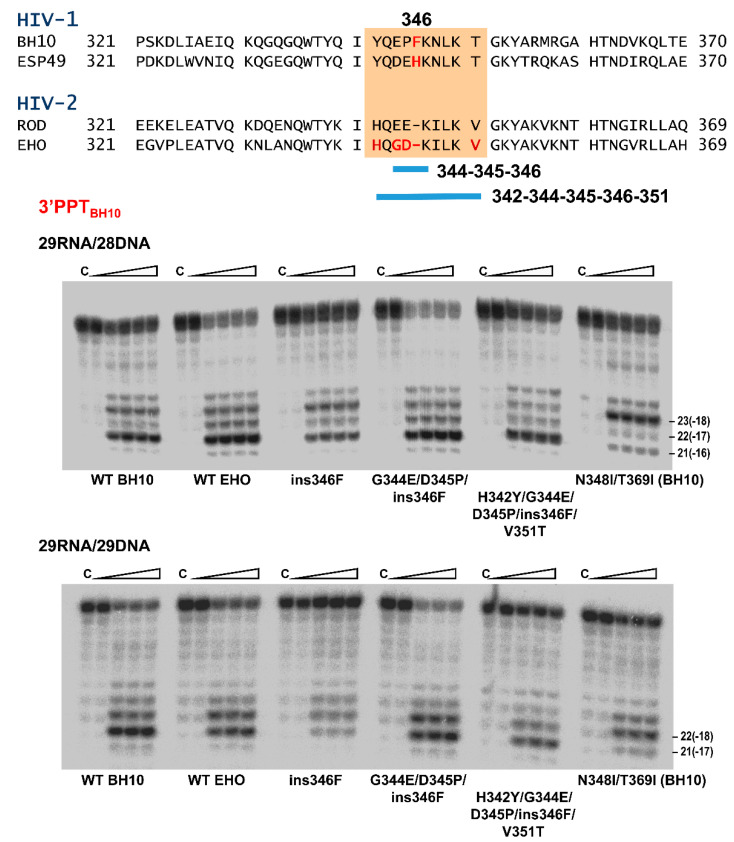
Effect of amino acid substitutions in the connection subdomain of HIV-2_EHO_ RT on the RNase H cleavage window. Amino acid sequence differences between representative HIV-1 and HIV-2 RTs are shown above. Sequence differences at positions 342-351 (HIV-1 RT numbering) are highlighted in an orange background. Gels below show representative RNase H cleavage assays carried out with 29RNA/28DNA and 29RNA/29DNA hybrids mimicking sequences found in the 3′PPT of HIV-1_BH10_ (see Figure 4A for details). Assayed mutants were obtained in the HIV-2_EHO_ RT background, except in the case of N348I/T369I which was made in the HIV-1_BH10_ RT. Reactions were carried out in the presence of RT and template-primer at concentrations of 125 nM and 25 nM, respectively. RT and template-primer were pre-incubated for 5 min at 37 °C, and aliquots were withdrawn at 0, 20, 40 and 180 s after the addition of magnesium and heparin. Lane C shows control experiments including heparin in the pre-incubation buffer.

In order to gain further insight into the molecular determinants of RNase H cleavage specificity at the PPT/U3 junction, we obtained mutants of HIV-2_EHO_ RT with substitutions around position 346. We concentrated on this particular region first, because it has a deletion of one amino acid found in HIV-2 but not in HIV-1 RTs; and second, because HIV-2 RTs contain Ile348 at the equivalent position of Asn348 in HIV-1 RTs (Figure 8, upper panel). In addition, amino acid sequences of HIV-1 and HIV-2 RTs are rather different in the vicinity of position 348 (about 50% identity). RNase H cleavage assays with 29RNA/28DNA and 29RNA/29DNA complexes representing the 3′PPT of HIV-1_BH10_ showed a reduced cleavage preference of the WT HIV-2_EHO_ RT for the G*A cleavage at PPT/U3 site rendering the 21-nt RNA product. In addition, this enzyme (as shown above for WT HIV-2_ROD_ RT; Figure 6) produced higher amounts of the 22-nt product in comparison with the WT HIV-1_BH10_ RT.

A step-wise approach was taken to determine whether the sequence around the one-amino acid deletion in HIV-2 RTs had an effect on the RNase H cleavage window and PPT removal specificity. First, we introduced an insertion of Phe or His (as found in HIV-1_BH10_ and HIV-1_ESP49_ RTs, respectively) at position 346 of HIV-2_EHO_ RT. Using the 29RNA/28DNA as substrate we found that the mutant ins346F RT produced a slightly different cleavage pattern in comparison with the WT HIV-2_EHO_ enzyme. A higher proportion of the 23-nt product was detected with ins346F RT, resembling the pattern obtained with WT HIV-1_BH10_ RT (Figure 8). However, differences between ins346F and WT HIV-2_EHO_ and HIV-1_BH10_ RTs were almost negligible with the 29RNA/29DNA substrate. The insertion of His instead of Phe had no effect on any of the analyzed cleavage patterns, independently of the PPT complexes used in the experiments (Supplementary Figure S3).

As shown in Figure 8, amino acid sequence alignments of HIV-1 and HIV-2 RTs reveal additional differences around the deletion that could have an impact on their correct positioning of RNA/DNA hybrids on the nucleic acid binding cleft. Therefore, we obtained a mutant HIV-2_EHO_ RT with the amino acid sequence Glu-Pro-Phe found at positions 344–346 of HIV-1_BH10_ RT instead of the Gly-Asp residues found in HIV-2_EHO_ RT. This engineered mutant, designated as G344E/D345P/ins346F, showed cleavage patterns identical to those obtained with WT HIV-2_EHO_ RT (Figure 8) indicating that introduced changes had a relatively small effect on the location of the susceptible PPT sequence. In contrast, two additional mutations at positions 342 and 351 rendered an HIV-2 RT with the capacity to complete the processing of PPT/U3 junction, even in conditions were the distance between the DNA polymerase and RNase H domain was reduced to about 16 nucleotides (i.e., with template-primer 29RNA/28DNA). These results are consistent with a critical role of the loop extending between β-sheets 17 and 18 (residues 342–350) in the connection subdomains of HIV RTs.

## 4. Discussion

The amino acid substitution N348I at the HIV-1 RT connection subdomain has a detrimental effect on RNase H activity [17,22,45] while reducing the association rate of RT and nevirapine [46]. These effects could be related to the altered positioning of the template-primer in the nucleic acid binding cleft of the RT. This interaction is most critical when involving RNA/DNA complexes containing a PPT sequence in the RNA strand. In agreement with this proposal, Biondi et al. showed that nevirapine enhanced PPT removal during initiation of plus-strand DNA synthesis, while the N348I mutation reverted this effect [30]. The suppressive effects of N348I were found to be stronger in the presence of T369I [17]. Unlike nevirapine and efavirenz, second-generation NNRTIs such as rilpivirine and etravirine had a small effect on the RNase H-mediated cleavage of the PPT/U3 junction by the WT HIV-1_BH10_ RT [17]. Interestingly, the results shown above reveal that doravirine, the most recently approved NNRTI, stimulates PPT removal to levels similar to those shown by nevirapine, and like in the case of this drug these effects can be suppressed by the combination of N348I and T369I.

Approval of doravirine was based on its improved efficacy, pharmacokinetics and safety profile compared with efavirenz, and its limited cross-resistance with rilpivirine and etravirine [47]. The highest levels of doravirine resistance (fold-change >100) have been associated with amino acid substitutions V106A/G190A/F227L, E138K/Y181C/M230L and Y188L (alone or in combination with K103N or V106I) [48]. However, doravirine appears to be more efficient than efavirenz and/or rilpivirine in suppressing resistance breakthrough of HIV-1 strains with the common NNRTI resistance-associated mutations such as K103N, Y181C or K103N/Y181C [49]. The effectiveness of doravirine in suppressing transmitted drug resistance mediated by K103N was crucial for its approval. From a structural point of view, doravirine is more rigid than rilpivirine and etravirine, but still maintains a certain degree of conformational flexibility upon binding to the HIV-1 NNRTI binding pocket [50]. It is possible that torsional constraints in the binding pocket restrict conformational changes in the connection subdomain that could facilitate the repositioning of the RNase H catalytic site and promote cleavage at the PPT/U3 junction as found in our assays (Figure 2). In this scenario connection subdomain mutations N348I/T369I appear to play a critical role by reducing RNase H-mediated cleavage of PPT-containing RNA strands (Figure 3).

In agreement with our previous report showing that NNRTI binding was required to produce an increase in PPT removal [17], integrase inhibitors (used as control in our experiments) had no effect on PPT/U3 cleavage. Recently, Malet et al. reported the emergence of an HIV-1 strain resistant to dolutegravir that contained a cluster of mutations in its 3′PPT, after successive passages of the virus in cell culture in the presence of the drug [51]. Moreover, in a subsequent clinical study, 3′PPT mutations were detected in one HIV-1-infected individual with virological failure during dolutegravir maintenance monotherapy [52]. Although the resistance mechanism involved remains unknown, Das and Berkhout speculated with the possibility that PPT mutations would affect cleavage efficiency and specificity at the PPT/U3 junction, modifying the 5’ and 3′ ends of the proviral DNA, required for its integration in the host cell chromosome [53]. However, the integrity of the long-terminal-repeat (LTR) ends was demonstrated by deep sequencing, in MT-4 cells infected with 3′PPT-mutated viruses [54]. Our results showing that integrase inhibitors have no effect on PPT removal also suggest that the selection of PPT-mutated viruses is not related to the initiation of plus-strand DNA synthesis.

Impaired PPT processing by the N348I/T369I HIV-1_BH10_ RT shown in our experiments could be attributed to its inability to locate the G*A site of the PPT/U3 junction at the RNase H active site, when the RT is bound in a polymerase-dependent mode (i.e., with 3′ end of the DNA at the polymerase active site). Molecular dynamics studies have shown that N348I/T369I did not induce any significant structural change, although those mutations modulate conformational dynamics by enhancing the rigidity of the connection subdomain due to the influence of neighboring hydrophobic residues that restrict the mobility of the RNase H domain [55]. Our findings demonstrate the dominant effect of both mutations in the presence of NRTI and NNRTI resistance mutations while other polymorphisms found in viruses selected under therapeutic pressure and not tested previously (at positions 399 and 400) had minor effects on the RNase H cleavage patterns obtained with N348I RT. The dominance of N348I could be reflected in a loss of viral fitness, that could prevent or delay the emergence of drug resistance under certain conditions. Thus, HIV-1 replication assays revealed that amino acid substitutions N348I or M184V/N348I decreased the replication capacity of viruses carrying E138K in their RT, a mutation that confers resistance to rilpivirine and etravirine [56]. Therefore, in this context, N348I could also delay the emergence of NNRTI resistance.

**Figure 9:**
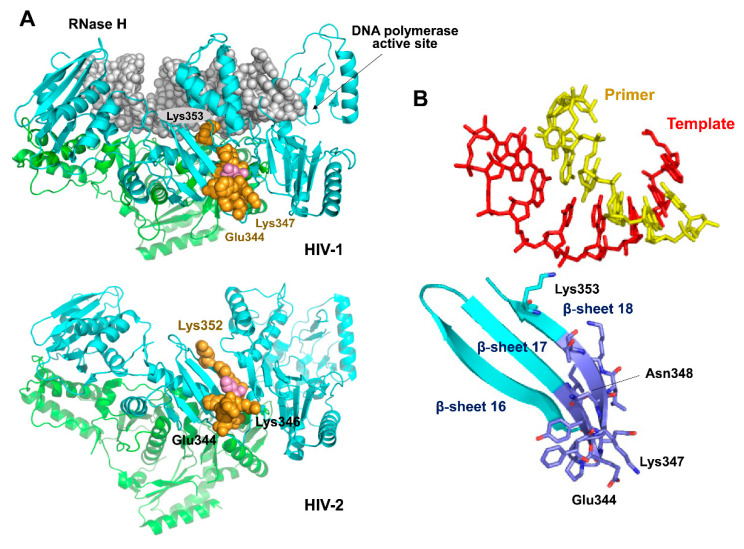
Location and structural context of Asn348 in HIV-1 and HIV-2 RTs. (**A**) Structure of a ternary complex of WT HIV-1 group M/subtype B RT bound to an RNA/DNA template-primer (upper panel) and unliganded HIV-2_ROD_ RT (lower panel). The position of the loop connecting β-sheets 17 and 18 in the large subunit of the heterodimer is indicated with a CPK model (orange spheres). Asn348 is shown in light magenta. (**B**) Close-up showing the location of relevant residues at the interaction site of Lys353 and the template strand of the RNA/DNA hybrid. Images were obtained with Pymol and the coordinates were taken from Protein Data Bank files 4B3P (HIV-1 RT) and 1MU2 (HIV-2 RT).

Despite showing important amino acid sequence differences around position 348 in the connection subdomain, HIV-1 and HIV-2 RTs were able to cleave PPT-containing RNA/DNA substrates in an efficient and specific manner, giving consistent but slightly different patterns with each PPT sequence. Interestingly, substituting the amino acid sequence found in HIV-1_BH10_ RT at positions 342–351 for the equivalent residues found in HIV-2_EHO_ RT increased PPT processing specificity, revealing that the connecting loop and β-sheets 17 and 18 (and including Asn348) were critical for maintaining the proper distance between the DNA polymerase and RNase H active sites of the RT (Figure 9). The analysis of crystal structures of binary and ternary complexes of HIV-1 RT bound to RNA/DNA and DNA/DNA template-primers shows that the side chain of Lys353 of p66 interacts with the template strand (RNA or DNA) [11,29,36,43,44]. In contrast, Lys353 and residues in its vicinity are located away from the nucleic acid binding cleft in the 51-kDa subunit of HIV-1 RT.

Proper β-sheet packaging is important to maintain the proper orientation of Lys353 and binding of the RNA template. The N348I substitution is likely to affect interactions involved in β-sheet packaging, while side-chains of Glu344 and Lys347 are exposed to the solvent. Although crystal structures of binary and ternary complexes of HIV-2 RT are still not available, the structure of the apoenzyme [57] reveals a similar conformation at the equivalent connecting loop and β-sheets 17 and 18 (Figure 9). Our mutagenesis experiments with HIV-2_EHO_ RT showed negligible effects on RNase H cleavage specificity when substituting residues at the connecting loop (e.g., G344E/D345P/ins346F). However, a measurable change in RNase H cleavage window was observed when additional substitutions were introduced at positions 342 and 351, expected to affect the packaging of β-sheets 17 and 18.

In summary, our results underscore the effect of some NNRTIs, most notably, nevirapine and doravirine on the initiation of plus-strand DNA synthesis, and the role of β-structures 17 and 18 in facilitating the proper positioning of the RNA template. Interestingly, Zhang et al. have reported that an investigational NNRTI designated as SJP-L-5 (methyl 6-[4-(dimethylamino)phenyl]-7-methoxy-1,3-benzodioxole-5-carboxylate) was a preferential inhibitor of PPT-primed plus-strand DNA synthesis (EC_50_ = 13.4 µM) over tRNA-primed minus-strand DNA synthesis (EC_50_ > 3.6 mM) [58]. This antiviral effect was attributed to impaired nuclear import of proviral DNA and its subsequent integration. However, using conventional assays, the authors did not observe any RNase H inhibitory effect of SJP-L-5 or nevirapine. Nevertheless, it is still possible that SJP-L-5 could affect the dynamics of the RT connection subdomain upon binding to the NNRTI binding site. In addition, long range interactions between RNase H and DNA polymerase domains in the RT have been recognized as important contributors to the effectiveness of antiretroviral therapy while affecting phenotypic susceptibility to NRTIs [27,28]. In this scenario, further studies on the role of the connection subdomain in reverse transcription and its potential as a druggable target in HIV-1 RT, including in vitro studies showing how connection subdomain mutations could influence viral fitness and intracellular reverse transcription, will hopefully provide novel clues in the design of innovative strategies for controlling HIV replication.

## Data Availability

The data presented in this study are available on request from the corresponding author.

## Supplementary Materials

The following are available online at https://www.mdpi.com/1999-4915/13/1/131/s1, Table S1: DNA oligonucleotides used in mutagenesis reactions, Figure S1: Template-primers used in RNase H cleavage window assays representing PPTs found in HIV-2 ROD and EHO strains, Figure S2: RNase H cleavage specificity of WT HIV-1_BH10_, HIV-2_ROD_ and HIV-2_EHO_ on representative HIV-2 PPTs, Figure S3: Effect of one-amino-acid insertions in the connection subdomain of HIV-2_EHO_ on the RNase H cleavage window.
