# Supplementary material for: Analysis and Molecular Determinants of HIV RNase H Cleavage Specificity at the PPT/U3 Junction
Source: Viruses. 2021 Jan 18;13(1):131. doi: 10.3390/v13010131 (PMC7831940; doi:10.3390/v13010131)
Supplement: Supplementary file 1 [file viruses-13-00131-s001.pdf]

Supplementary Information

Analysis and molecular determinants of HIV RNase H cleavage specificity at the PPT/U3 junction

Mar Álvarez, Enrique Sapena-Ventura, Joanna Luczkowiak, Samara Martín-Alonso & Luis Menéndez-Arias*

Centro de Biología Molecular Severo Ochoa (Consejo Superior de Investigaciones Científicas & Universidad Autónoma de Madrid), Madrid, Spain

* Corresponding author. E-mail: lmenendez@cbm.csic.es

Supplementary Table S1. DNA oligonucleotides used in mutagenesis reactions.[a]

RT	Mutants	Sequences
HIV-1$_{BH10}$	M184I	5′ TATCTATCAATACATCGATGATTTGT 3′<br>5′ ACAAATCATCGATGTATTGATAGATA 3′
	M184V	5′ TATCTATCAATACGTGGATGATTTGT 3′<br>5′ ACAAATCATCCACGTATTGATAGATA 3′
	E399D	5′ GGAAACATGGGATACATGGTGGACAGAGTATTGG 3′<br>5′ CCAATACTCTGTCCACCATGTATCCCATGTTTCC 3′
	E399G	5′ CAAAAGGAAACATGGGGAACATGGTGGACAGAG 3′<br>5′ CTCTGTCCACCATGTTCCCCATGTTTCCTTTTG 3′
	T400A	5′ GGAAACATGGGAAGCATGGTGGACAGAGTATTGG 3′<br>5′ CCAATACTCTGTCCACCATGCTTCCCATGTTTCC 3′
	T400S	5′ GGAAACATGGGAATCATGGTGGACAGAGTATTGG 3′<br>5′ CCAATACTCTGTCCACCATGATTCCCATGTTTCC 3′
HIV-2$_{EHO}$	H342Y	5′ GACCTATAAAATTTATCAGGAGCCTTTC 3′<br>5′ GAAAGGCTCCTGATAAATTTTATAGGTC 3′
	G344E/D345P	5′ AAAATTCATCAGGAGCCTTTCAAAATCCTGA 3′<br>5′ TCAGGATTTTGAAAGGCTCCTGATGAATTTT 3′
	Δ346F	5′ CATCAGGGCGATTTCAAAATCCTGAAAGTGGGT 3′<br>5′ ACCCACTTTCAGGATTTTGAAATCGCCCTGATG 3′
	Δ346H	5′ CATCAGGGCGATCATAAAATCCTGAAAGTGGGT 3′<br>5′ ACCCACTTTCAGGATTTTATGATCGCCCTGATG 3′
	V351T	5′ CAAAATCCTGAAAACGGGTAAATACGCAA 3′<br>5′ TTGCGTATTTACCCGTTTTCAGGATTTTG 3′

[a] Left column indicates the mutation(s) or insertions introduced in the RT.

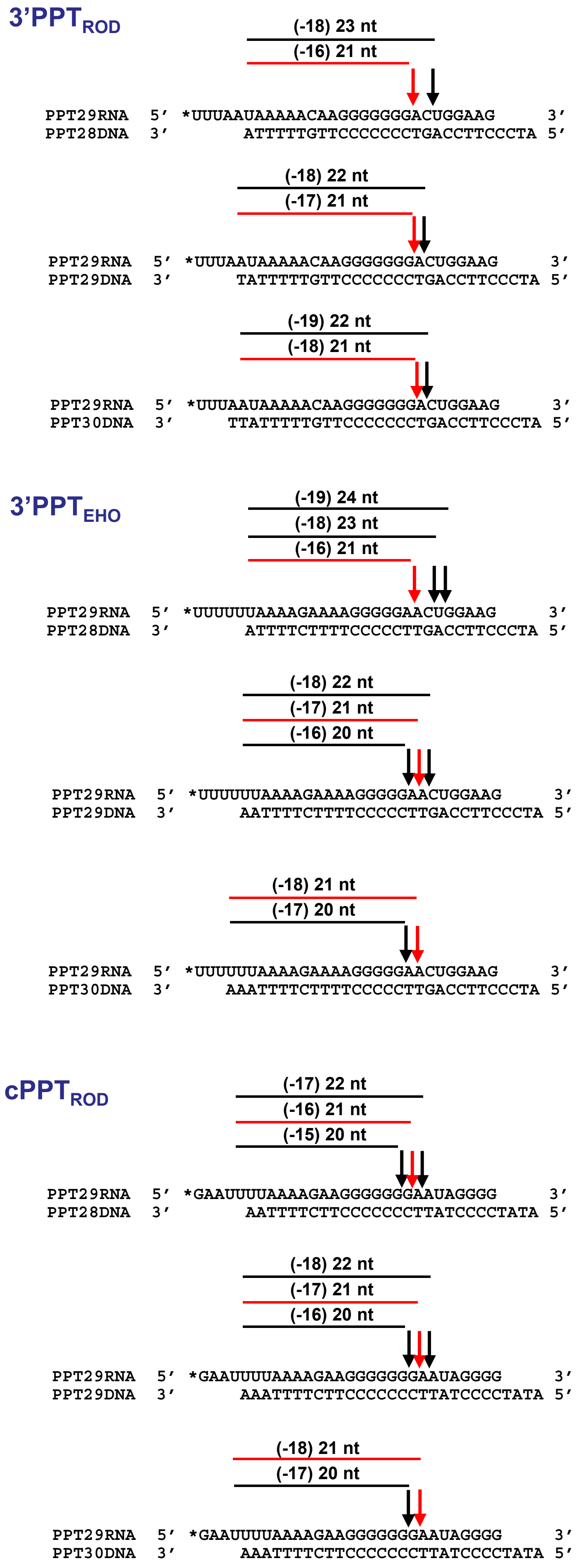

Figure S1. Template-primers used in RNase H cleavage window assays representing PPTs found in HIV-2 ROD and EHO strains. Major cleavage sites observed are indicated above the RNA sequences.

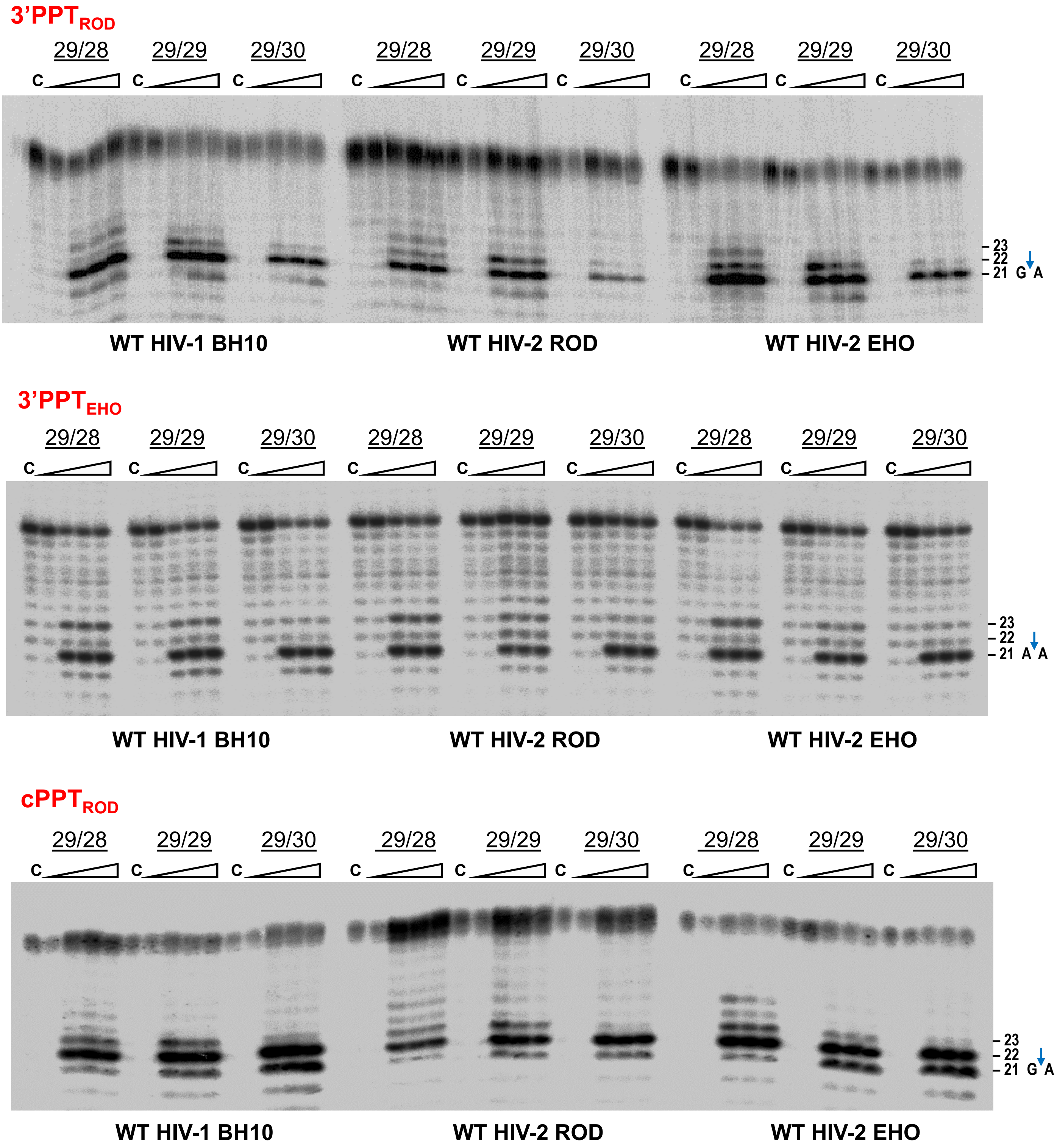

Figure S2. RNase H cleavage specificity of WT HIV-1$_{BH10}$, HIV-2$_{ROD}$ and HIV-2$_{EHO}$ on representative HIV-2 PPTs. Reactions were carried out in the presence of enzyme and template-primer at concentrations of 125 nM and 25 nM, respectively. RT and template-primer were preincubated during 5 min at 37 ºC, and aliquots were withdrawn at 0, 20, 40 and 180 s after the addition of magnesium and heparin. Lane C shows control experiments including heparin in the preincubation buffer. Numbers above indicate the template-primer used in each experiment: 29/28, 29/29 and 29/30 for 29RNA/28DNA, 29RNA/29DNA and 29RNA/30DNA hybrids, respectively (see Supplementary Figure S1 for sequence details).

```
                                                       346
HIV-1
BH10    321   PSKDLIAEIQ KQGQGQWTYQ IYQEP F KNLK TGKYARMRGA HTNDVKQLTE 370
ESP49   321   PDKDLWVNIQ KQGEGQWTYQ IYQDE H KNLK TGKYTRQKAS HTNDIRQLAE 370

HIV-2
ROD     321   EEKELEATVQ KDQENQWTYK IHQEE - KILK VGKYAKVKNT HTNGIRLLAQ 369
EHO     321   EGVPLEATVQ KNLANQWTYK IHQGD - KILK VGKYAKVKNT HTNGVRLLAH 369
```

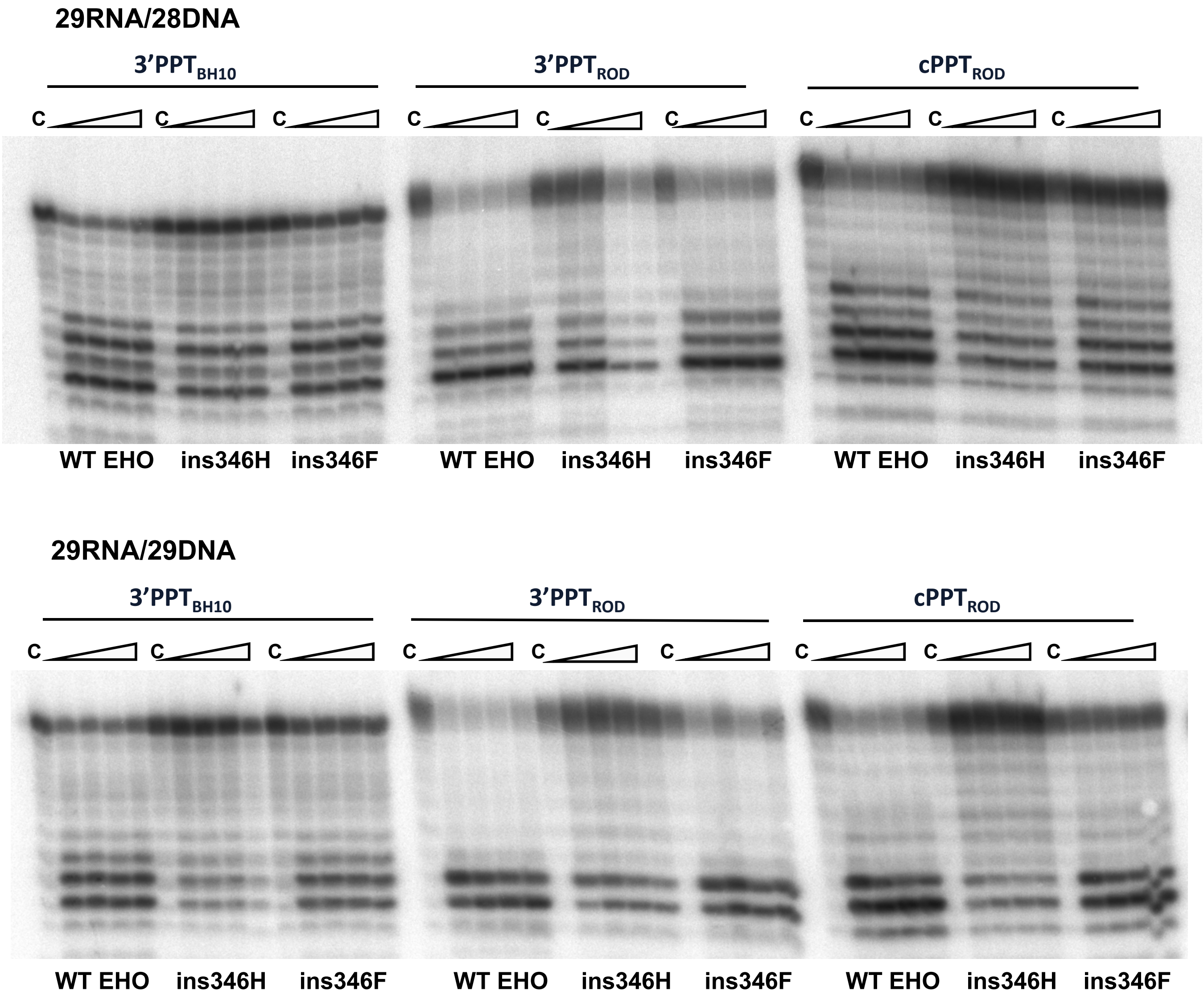

Figure S3. Effect of one-amino-acid insertions in the connection subdomain of HIV-2_{EHO} on the RNase H cleavage window. Amino acid sequence differences between representative HIV-1 and HIV-2 RTs are shown above. Gels below show representative RNase H cleavage assays carried out with 29RNA/28DNA and 29RNA/29DNA hybrids mimicking sequences found in representative PPTs of HIV-1 and HIV-2 strains (see Figure 4A and Supplementary Figure S1 for details). Reactions were carried out in the presence of RT and template-primer at concentrations of 125 nM and 25 nM, respectively. RT and template-primer were preincubated during 5 min at 37 ºC, and aliquots were withdrawn at 0, 20, 40 and 180 s after the addition of magnesium and heparin. Lane C shows control experiments including heparin in the preincubation buffer.
